# Autonomous Quadcopter Landing on a Moving Target

**DOI:** 10.3390/s22031116

**Published:** 2022-02-01

**Authors:** Alvika Gautam, Mandeep Singh, Pedda Baliyarasimhuni Sujit, Srikanth Saripalli

**Affiliations:** 1 Department of Mechanical Engineering, Texas A&M University, College Station, TX 77840, USA; alvikag@tamu.edu; 2 Robotic Institute, Carnegie Mellon University, Pittsburgh, PA 15213, USA; msingh2@andrew.cmu.edu; 3 Department of Electrical Engineering and Computer Science, IISER Bhopal, Bhauri, Bhopal 462066, India; sujit@iiserb.ac.in

**Keywords:** UAV, landing, guidance, vision

## Abstract

Autonomous landing on a moving target is challenging because of external disturbances and localization errors. In this paper, we present a vision-based guidance technique with a log polynomial closing velocity controller to achieve faster and more accurate landing as compared to that of the traditional vertical landing approaches. The vision system uses a combination of color segmentation and AprilTags to detect the landing pad. No prior information about the landing target is needed. The guidance is based on pure pursuit guidance law. The convergence of the closing velocity controller is shown, and we test the efficacy of the proposed approach through simulations and field experiments. The landing target during the field experiments was manually dragged with a maximum speed of 0.6 m/s. In the simulations, the maximum target speed of the ground vehicle was 3 m/s. We conducted a total of 27 field experiment runs for landing on a moving target and achieved a successful landing in 22 cases. The maximum error magnitude for successful landing was recorded to be 35 cm from the landing target center. For the failure cases, the maximum distance of vehicle landing position from target boundary was 60 cm.

## 1. Introduction

Quadrotors are ubiquitous in numerous applications such as surveillance [1], agriculture [2,3], and search and rescue operations [4] because of their agile maneuvering, ability to hover, and ease of handling. During an autonomous aerial mission, landing of a quadrotor is the most critical task as any uncontrolled deviations can result in a crash. The task becomes even more challenging if unmanned aerial vehicle (UAV) has to track a moving ground target with minimum information while executing the landing maneuver simultaneously. This combined with time-critical requirements of certain applications and limited flight time of quadrotors makes accurate and timely completion of autonomous landing a challenging problem.

Autonomous landing of UAVs broadly involves two steps: (a) detection and/or knowledge of the landing pad (also referred to as the target) and (b) generation of motion control commands for the UAV to accurately land on the target. For the first component, i.e., target detection, landing using only GPS based target information is prone to errors of anywhere between 1 to 5 m [5,6] because of large covariance in measurements for localization. GPS is also unreliable in closed spaces and other GPS-denied environments. The use of onboard cameras and computer vision techniques for object tracking and localization is a popular way to overcome such shortcomings to enable autonomous landing not only on stationary but mobile targets as well [7,8,9]. Solutions for detecting the target using other sensors like light detection and ranging (LIDAR) [10], ultrasonic sensors, and IR sensors [11] are also available in the literature. Although interesting results were achieved, these approaches are not necessarily applicable to real-time outdoor applications involving moving targets. Both ultrasonic and IR sensors are sensitive to surface and environmental changes, and although LIDAR provides a very accurate estimate of distances, it is bulky and expensive for quadrotor applications.

Computer vision techniques to detect the landing target using onboard cameras was widely studied in literature [12,13,14,15,16,17]. Additionally, for landing on moving targets, a number of works have explored coordinated landing with active communication between the UAV and the target [18,19,20]. In this work, we do not rely on any communication between the UAV and the target to coordinate a landing maneuver.

Image-based visual serving is a popular approach for vision-based precision landing [21,22,23]. However, it relies on the landing target being visible throughout the task since it relies solely on visual information. To deal with scenarios such as intermittent loss of landing target, model-based approaches [24,25,26] were proposed to predict the trajectory of the landing target. Alternative solutions include attachment of additional sensors on the moving target (e.g., GPS receivers [27]). Additionally, some of the previously mentioned solutions rely on external computation such as communication between the UAV and a ground station for state estimation [25] and a Vicon system to deal with cases of no target detection [22], which is a limitation toward fully autonomous operation. To know the target location for plotting purposes, we mount a GPS on the target to record the trajectory of the target. We do not rely on an external motion capture system or active communication of information from target such as target location, velocity etc., to carry out the landing maneuver.

For the second component of autonomous landing, that is, generation of motion control commands based on target information, we will focus on the discussion of vision-based control techniques.

A large body of literature focused on traditional control theory approaches for landing [28] such as feedback linearization [29], backstepping control [30], mixed $H_{2}$–$H_{\infty }$ [31] and fuzzy logic controllers [32,33,34,35]. Although these methods were extensively analyzed for stability, most of the testing was limited to simulation results.

**Figure 1:**
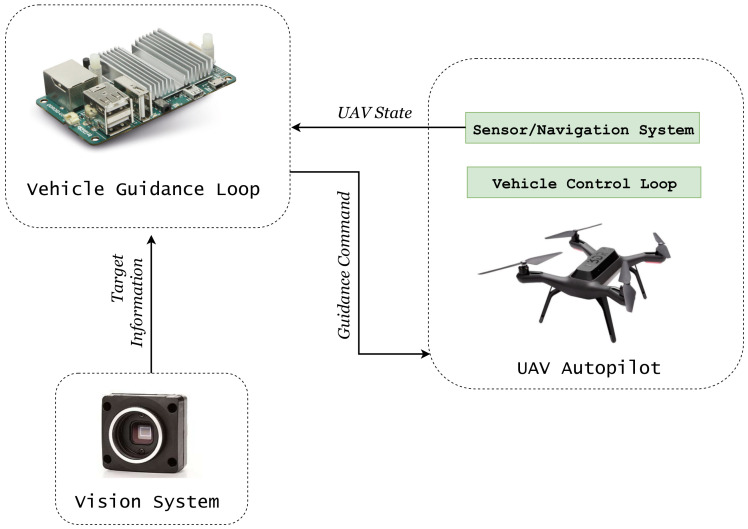
Block diagram of a vision-assisted guidance landing system.

Application of missile guidance laws to autonomous landing drew significant attention in the literature [36]. This typically involves the use of successive loop closure [37], which separates guidance and control into two separate loops, as shown in Figure 1. Guidance, in general, refers to the determination of heading from the vehicle’s current location to a designated target. The guidance commands are generated by an outer loop based on the target information. An inner loop controls the UAV attitude according to the generated guidance commands. It is desirable to use this approach as it is more flexible and offers finer control on each module without interfering with the working of inner proportional integral derivative (PID) control loops of the UAV and making it more flexible to integrate with different methods of target sensing .

Popular guidance approaches for landing include pursuit guidance laws such as nonlinear pursuit [38] and pseudopursuit [39], proportional navigation [27], and $t_{go}$ guidance [40]. Although, guidance-based approaches are simple to integrate with vision in the loop while offering a finer control on the UAV trajectory, most uidance-based landing techniques proposed in the literature were tested for fixed-wing UAVs [41]. On the other hand, vision-based guidance techniques for quadrotor landing were limited to vertical landing approach only [42]. In such approaches, a quadrotor tracks the target while moving along a linear path till a certain distance, and then descends vertically to land on the target. This can result in increased flight time during the landing process. In this paper, we propose to use a simultaneous “track and descend” approach instead of a vertical landing approach for time efficient landing on a moving target using pure pursuit guidance. One of the obvious challenges of simultaneous track and descend approach is a faster decrease in camera field of view, which can quickly result in loss of the landing target. This warrants the need for a finer control on the speed profile of the UAV such that the UAV approaches and descends fast enough (for a time-efficient landing), while ensuring that the target does not go out of the field of view. To this end, we integrate the pure pursuit (PP) guidance law with a closing velocity controller.

Next, we list the contributions of this work.

### Contributions

This work builds upon the authors’ previous work [43,44]. The work in [43] is validated in outdoor experiments for a stationary target and robustness of the approach for target tracking in the presence of environmental disturbances is presented in [44]. In this work, we extend the approach to moving targets. The major contributions of this work are as follows:

- A fully autonomous PP-based guidance implementation with vision in the loop for time-efficient landing on moving targets, which descends and tracks the target simultaneously as opposed to track and vertical land approaches. The guidance approach is integrated with a log-polynomial function based closing velocity controller.
- We add AprilTags to our landing target design and use AprilTag detection in addition to color-based object segmentation for detecting the landing pad. The proposed landing pad consists of AprilTags of multiple sizes and a logic is developed that switches between the two detection methods (blob detection and AprilTag detection) to accurately detect the landing pad from varying altitudes. A Kalman filter is used for target state estimation.
- This work leverages the analysis of the controller’s parameter characterization based on different speed profiles presented in [45] for automatic initialization of the controller’s parameters, based on the initial estimate of target’s velocity. Thus, the approach does not rely on any prior knowledge of the target motion or trajectory.Also, the approach does not rely on any active sensor data being communicated from target to the aerial drone system about its state (position and velocity).
- We demonstrate the performance of the proposed guidance law through realistic simulations for different target speeds and trajectories.
- We evaluate the robustness of the approach through extensive real-world experiments on off the shelf 3DR solo quadrotor platform. We perform a total of 27 experiment runs, with scenarios consisting of straight line as well as random target trajectory along with scenarios of target occlusion.
- Based on the findings of experiment results, we derive a a lower bound for vertical velocity of the UAV using the proposed controller so as to consistently maintain the visibility of the target so as to extend the approach for higher speed landing targets.

## 2. Materials and Methods

### 2.1. Landing Pad Detection Using Vision

**Figure 2:**
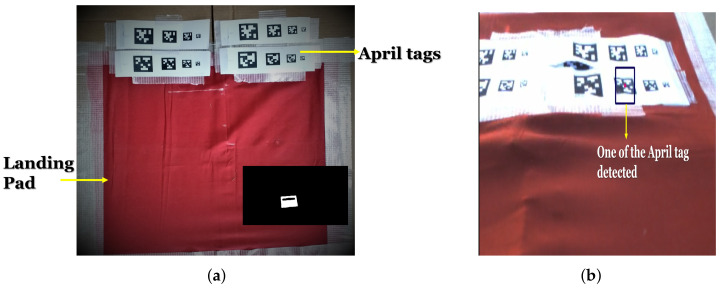
(**a**) Target setup for autonomous landing. Inset figure shows extracted contour of red blob (**b**) One of detected AprilTags.

Accurate and fast detection of the landing pad is essential. There are several visual patterns that can be used for a landing pad with different computational complexity [28]. In this work, we assume that the landing target is of known color (red) and no distractor targets are present in the vicinity. As the computational unit on board the vehicle is small, we use color segmentation and AprilTags [46] as the mechanism to detect the landing pad. Both these techniques have low computational complexity, and hence, can detect the pad at a high frame rate. Additionally, this approach does not require a particularly high image resolution, unlike approaches that require small object detection and accurate shape detection. Figure 2a shows the landing pad in the form of a red colored rectangle, with 16 AprilTags of varying sizes covering a portion of this red target. The red portion of the landing pad is detected using blob detection algorithm. The obtained RGB image in every frame is converted to HSV color space due to its robustness to change in lighting conditions. Hue defines the dominant color of an area, saturation measures the colorfulness of an area in proportion to its brightness, and the value is related to the color luminance.

Additionally, the original RGB image is also converted to YCrCb color space. In this space, color is represented by *luma*, constructed as a weighted sum of RGB values, and two color difference values Cr and Cb that are formed by subtracting *luma* from RGB red and blue components. A thresholding operation to detect red colored pixels was performed individually on both HSV and YCrCb color space frames.

For color segmentation, HSV performs better than RGB color space under varying lighting conditions. However, due to the nonlinear transformation involved in obtaining the HSV components from RGB results in nonremovable singularities (for black, white, and gray colors), which can cause issues. On the other hand, YCrCb components are obtained by a linear transformation of the RGB channels that is simpler, but due to the linear transformation there is a higher correlation between the component channels that can lead to false detection.

Through experiments, we empirically found that a mask obtained by combining both the HSV and YCrCb color segmented masks gave the best results for target detection in outdoor environment with nonuniform lighting conditions. Therefore, a *bitwise OR* operation was performed on the two obtained red pixel masks, and morphological operations were applied to remove the noise while preserving the final mask boundaries. Erosion was performed using a 8×8 rectangular kernel, followed by dilation using a 3×3 rectangular kernel. The red blob is detected by extracting the largest contour in the scene, as shown in the inset figure of Figure 2a.

During the terminal landing phase, camera footprint (m${}^{2}$ area visible to camera at a specific altitude) is small, and hence, only a part of the target is visible or the complete frame is filled with the blob. At this point, our vision algorithm switches from detecting blobs to detecting AprilTags. The altitude at which this switching happens was 1 m in our experiments. Figure 2b shows the AprilTag selected by the vision algorithm. Such a landing pad design ensures flexibility and detectability from high as well as very low altitudes. AprilTags were just used for detecting the target center below a threshold altitude (similar to red blob detection at higher altitudes). We did not use AprilTags for pose estimation as that can be computationally expensive. The decision-making logic to switch between blob and AprilTag detection and AprilTag selection logic is explained in detail in Section 4. Additionally, we employ a target estimator to deal with cases of partial/full target occlusion as discussed next for efficient tracking of the target.

#### Target State Estimation Using Kalman Filter

During the landing process, the target may be partially or fully occluded, or may move out of the field of view. Hence, we use Kalman Filter (KF) to estimate the target parameters. The detection algorithm determines the *x* and *y* centroid pixel coordinates of the target, which is given as the input to the KF. The target is modeled in discrete time as,

(1) $$\begin{array}{ccc} X_{t}\left(n\right) & = & F_{t}X_{t}\left(n-1\right)+w\left(n\right), \end{array}$$

where, w(n) is zero mean Gaussian process noise and the subscript *n* denotes the current time and n−1 denotes the previous time instant. $X_{t}$ is the state vector comprising of target characteristics: centroid pixel coordinates ($x_{t}$, $y_{t}$), rate of change of pixel coordinate positions (${\dot{x}}_{t}$,${\dot{y}}_{t}$), dimensions of the detected target contour (width$\left(w_{c}\right)$, height$\left(h_{c}\right)$). The approach does not rely on distinguishing between width and height of the contour and instead utilizes the product (width*height) of the detected contour to give an insight into the area of the blob, since sudden changes in area may imply a wrong measurement or a false positive. $F_{t}$ is the state-transition model that is applied to the previous state $X_{t}\left(n-1\right)$. We measure the position of the target in image frame using vision. Measurement at time *n* is given by,

(2) $$\begin{array}{ccc} Z_{t}\left(n\right) & = & H_{t}X_{t}\left(n\right)+v\left(n\right), \end{array}$$

where, $H_{t}$ is the measurement model and v(n) is zero mean Gaussian measurement noise in landing pad parameter measurements. Both process and measurement noise are modeled as white, zero mean Gaussian noise.

The final estimated parameters of the target from the predict and update steps of the KF are given as input to the guidance loop.

Although we do make an assumption about the landing target being of a known color (red), overall, our landing target detection algorithm is reasonably robust to small red blobs in the environment, as the detection algorithm looks for the biggest contour as the landing target. Additionally, any sudden change in contour size or target center is mitigated by the Kalman filter estimation.

### 2.2. Log Polynomial Velocity Controller for Autonomous Landing

The guidance objective is to land the vehicle smoothly on the target while persistently tracking it. In this work, we use PP for autonomous landing control. PP works on the principle of consistently aiming to align the follower vehicle’s (i.e., the UAV) velocity vector towards the target. The guidance command for simple pure pursuit is given as,

(3) $$\begin{array}{cc} a_{xy} & =V_{u}{\dot{\lambda }}_{xy}-K_{a}\left(\chi_{u}-\lambda_{xy}\right), \end{array}$$

where, $\chi_{u}$, $\chi_{t}$, $\lambda_{xy}$, and $R_{xy}$ represent the UAV course angle, target course angle, line-of-sight (LOS) angle, and the distance between target and the UAV (range), respectively. $V_{u}$ is the UAV velocity and $K_{a}$ is the gain.

The details of 2D PP guidance and its extension to 3D for application to a landing scenario are described in Appendix A.

For a safe and precise landing, closing velocity (velocity with which the tracking vehicle closes on to the target) should reduce asymptotically to zero. This condition is mathematically represented as,

(4) $$\begin{array}{c} \lim_{R\to 0}-\dot{R}\to 0. \end{array}$$

PP given in Equation (3) works under the assumption of constant UAV speed and thus does not control the UAV speed to drive the UAV target’s closing velocity to zero at the time of landing. To achieve the closing velocity requirement while landing in a time-efficient manner, we propose a log of polynomial function to control the vehicle speed. We integrate the speed controller with PP. The log of polynomial function is given as,

(5) $$\begin{array}{ccc} f(x) & = & N^{log_{n}\left(1+ax+bx^{2}+cx^{3}\right)}, \end{array}$$

(6) $$\begin{array}{ccc} N & = & V_{u}^{0}/V_{t}^{0}, \end{array}$$

(7) $$\begin{array}{ccc} x & = & R_{xy}/R_{xy}^{0} \end{array}$$

subject to,

(8) $$\begin{array}{ccc} 1+a+b+c & = & n, \end{array}$$

where *N* is the ratio of initial UAV to target velocity and *x* is defined as the ratio of the current range of the UAV to target to initial UAV target range. UAV velocity is varied as,

(9) $$\begin{array}{c} V_{u}=f\left(x\right)V_{t} \end{array}$$

where $V_{u}$ and $V_{t}$ are UAV and target velocity respectively. The variation of UAV velocity using a combination of pure pursuit and log polynomial speed controller assures the convergence of closing velocity to zero as the distance between UAV and the target (*x*) approaches zero during landing. Please refer to Appendix B for a complete proof of the same.

Besides achieving the desired closing velocity conditions, log of polynomial function has other desirable properties of the UAV flight as,

1. It has a slower decay for most of the flight, which makes sure that velocity of the UAV is significantly higher than the target.
2. Faster decay towards the end that quickly drives the closing velocity to minimum as the UAV approaches the target.

In 3D, $-{\dot{R}}_{xy}$, $\lambda_{xy}$ reflect the velocity with which the UAV approaches the target and its alignment with the target. $-\dot{R_{z}}$ reflects the velocity with which the UAV descends. To satisfy the terminal landing condition given by Equation (4), it is important to drive both $-\dot{R_{xy}}$ and $-\dot{R_{z}}$ to zero. As explained in Theorem A1, $-\dot{R_{xy}}$ is driven to zero by driving velocity of UAV close to velocity of target. Driving $-\dot{R_{z}}$ to zero at the time of landing implies,

(10) $$\begin{array}{c} \lim_{z\to 0}V_{z}\to 0. \end{array}$$

To achieve condition given in Equation (10), we use an alternative log polynomial function for altitude control and this velocity profile is given as,

(11) $$V_{z}=V_{z0}\log_{n}\left(1+ax+bx^{2}\right),$$

where *x* represents the ratio of current to initial altitude, and $V_{z0}$ represents the initial velocity in *z* direction.

Most of the guidance-based landing techniques presented in literature [27,38,39] meet only the terminal constraints of landing without offering any explicit control over the velocity profile of the throughout the landing process. Although time-based guidance approaches exist that provide flexibility in terms of velocity profile control [40], they are prone to replanning requirements in case of system delays.

**Figure 3:**
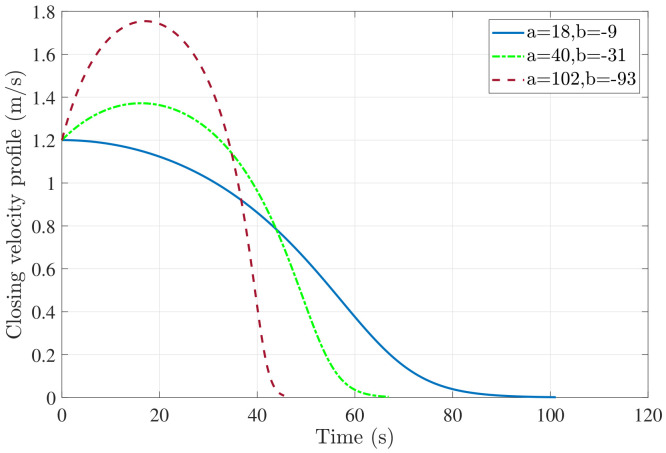
Effect of variation of (a,b) on closing velocity profile for moving target landing; $V_{u0}=2$ m/s, $V_{t}=1$ m/s and n=10.

Our approach is distance- instead of time-based and overcomes some of these challenges as the solution design offers a more flexible control. Figure 3 shows the effect on the variation of parameters (a,b) on the velocity profile. For the sake of simplicity, we show the variation for two control parameters of the log polynomial instead of three. By varying (a,b), we can control the landing velocity profile and various parameters like nature of the profile (initially accelerating or decreasing), maximum velocity attained, and time taken to land. Additionally, the theoretical analysis provided in [45] can be leveraged to serve as an automated lookup table to initialize as well as modify design parameters in different missions as well as during the same mission. This is a significant advantage as it not only provides a control on the terminal landing process but also allows to simultaneously incorporate multiple flight constraints (rate of velocity decay, maximum UAV, time to land) throughout the mission.

The decoupled nature of vision and control makes this approach simple and flexible to integrate with any distance based sensing modality. Further, the proposed controller allows for development of independent velocity control subsystems (Equation (9) for individual control of longitudinal and lateral velocity profiles based on target behavior), as well as ease of modification for vertical velocity profile control (11); therefore, it provides a practical as well as sound solution for real-time landing applications.

## 3. Simulation Results

We evaluate the performance of the developed vision-based PP guidance with log polynomial velocity controller using simulations on Microsoft Airsim and MATLAB, followed by experimental demonstration.

### 3.1. Simulation Setup

**Figure 4:**
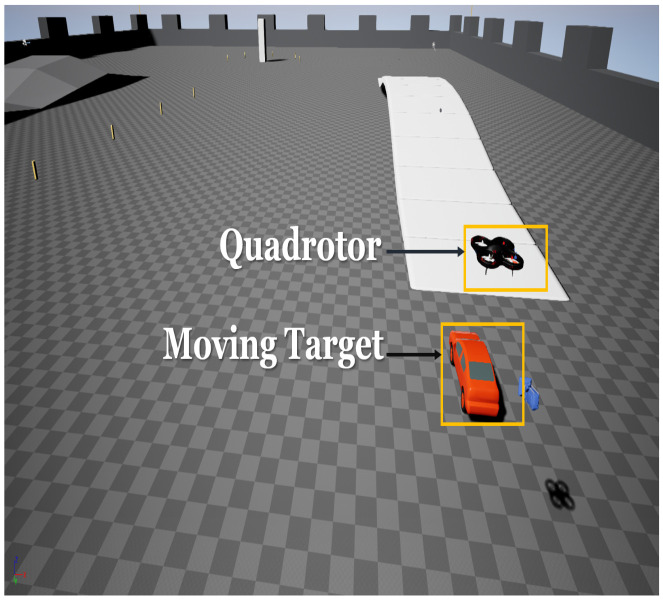
A snapshot of simulation setup consisting of airsim flying quadrotor vehicle and a ground vehicle as landing target.

To evaluate the proposed guidance-based controller, we use the Microsoft Airsim [47] simulator using Unreal Engine [48] as the base. The vehicle simulated within airsim has a camera model (orientation and direction can be specified) and sensor models such as accelerometer, gyroscope, barometer, magnetometer, and GPS. Onboard stream (of 640×480 resolution) of the downward camera was used for target detection. The landing target is in the form of a moving car simulated in unreal engine. We use the blob detection approach discussed in Section 2.1 to identify the landing target. Figure 4 shows a screenshot of the simulation environment consisting of the flying quadrotor vehicle modeled in Airsim and a ground vehicle, which served as the moving landing target.

We evaluated the proposed strategy through airsim simulations for a straight-line moving target for 3 different speed cases and a circular trajectory moving target for two different minimum turning radii. In each scenario, the quadrotor was initialized at an altitude of 20 m.

### 3.2. Landing on a Straight Line Moving Target

In this case, we evaluate the performance of the system when the target is moving in a straight line motion with a constant speed. Simulations with different target speeds of 1 m/s, 2 m/s and 3 m/s are carried out.

**Figure 5:**
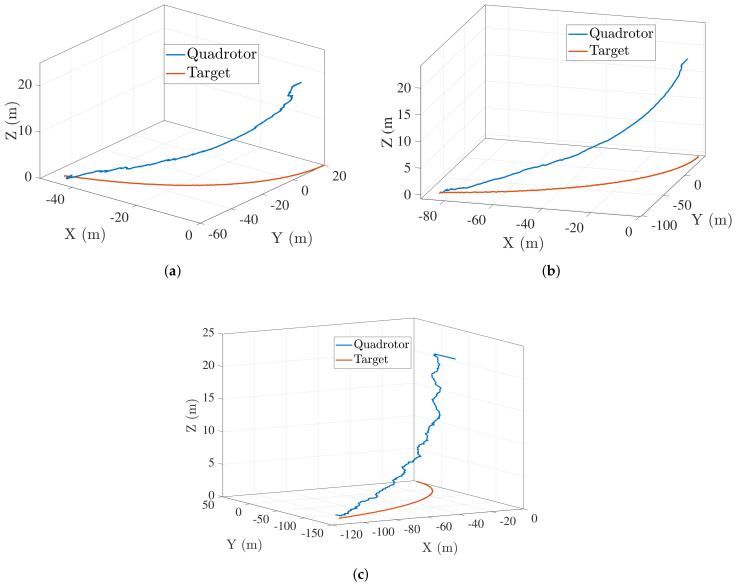
Landing trajectory followed by unmanned aerial vehicle (UAV) when ground vehicle moves in a near straight line trajectory at (**a**) $V_{t}=1$ m/s (**b**) $V_{t}=2$ m/s (**c**) $V_{t}=3$ m/s .

Figure 5a–c shows the trajectory followed by the quadrotor with the vision-based target input when the target is moving with a constant speed of 1 m/s, 2 m/s, and 3 m/s respectively. With vision in the loop, the quadrotor follows an efficient trajectory towards the target.

**Figure 6:**
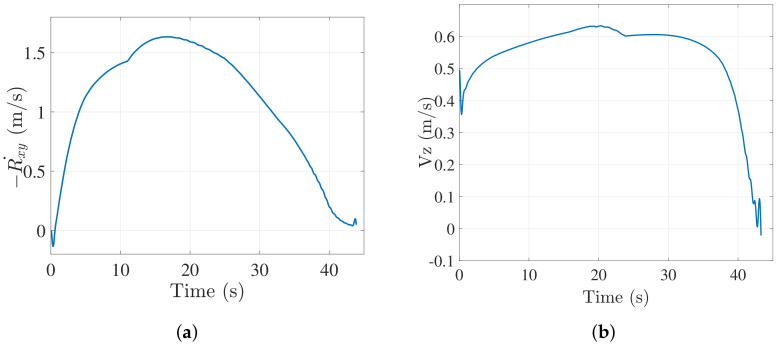
(**a**) Closing velocity profile (xy plane) of UAV when vehicle moves in a near straight line trajectory at 2 m/s. (**b**) Descend velocity of UAV when ground vehicle moves in a near straight line trajectory at 2 m/s.

Figure 6a,b show the closing velocity profile and the descent velocity of the UAV respectively for 2 m/s target speed case. It can be observed that both converge to zero as the vehicle lands on the target.

### 3.3. Landing on a Circular Trajectory Moving Target

In this case, we analyze the performance of the quadrotor landing when the target is moving in a circular trajectory of radii 5 and 15 m with a speed of 2 m/s.

**Figure 7:**
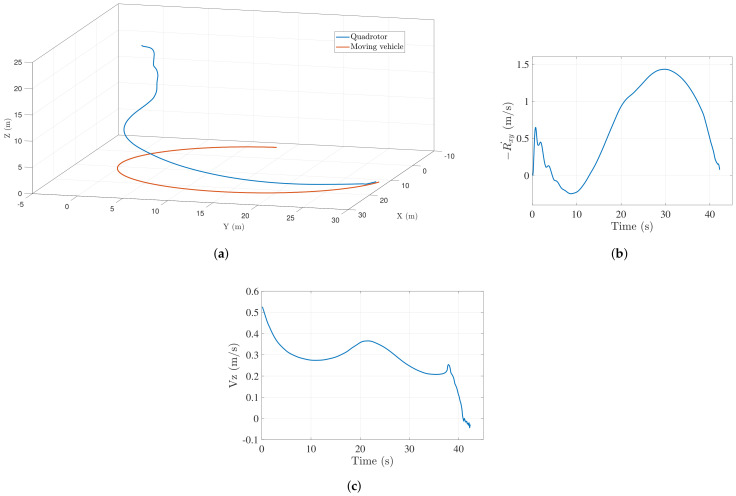
(**a**) Landing trajectory followed by UAV when ground vehicle moves along a circular path (15 m radius); (**b**) closing velocity profile (xy plane) of the UAV when ground vehicle moves along a circular path (15 m radius); (**c**) descend velocity of the UAV when ground vehicle moves along a circular path (15 m radius).

Figure 7a shows the trajectory followed by the vehicle to successfully land on the target for 15 m radius case. As observed from the figure, the quadrotor tracks the trajectory of the target vehicle and efficiently adjusts itself to follow along the same path and finally land on the target accurately.

**Figure 8:**
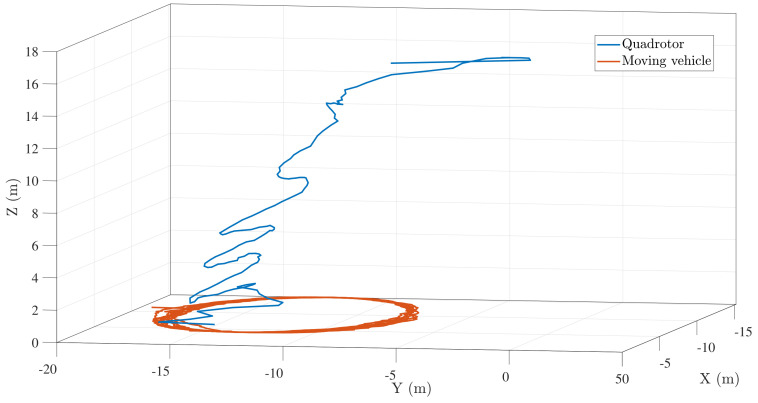
Landing trajectory followed by UAV when ground vehicle moves along a circular path (5 m radius).

Figure 7b,c show the closing velocity profile and the descent velocity of the UAV respectively. Both converge close to zero as the vehicle lands on the target. Figure 8 shows a similar trend of trajectory, followed by successful landing when the landing target was made to follow a circular path of a smaller radius (5 m approx.). Since the target maneuvers rapidly, the vehicle takes a longer duration to land in this case. For the 5 m radius case, the aerial vehicle descends slower than the straight line case since the longitudinal and lateral controllers have to keep the small radius target in the field of view.

### 3.4. Matlab Simulation Results: Landing with Noise in Target Information

In the previous case, it was assumed that accurate information about the target is available to the UAV. In the field, the received target information is often noisy. Therefore, to analyze the robustness of the landing controller simulations are performed with a noisy target model.

**Figure 9:**
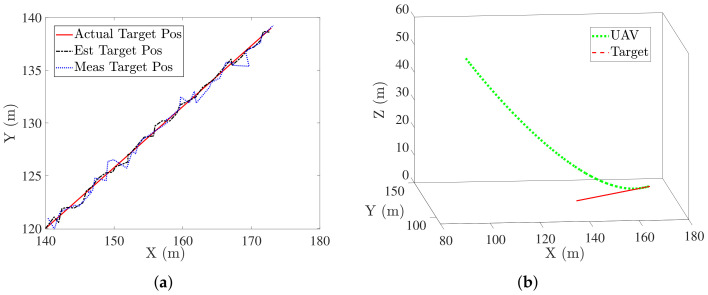
(**a**) Kalman filter performance for estimating target information (**b**) Trajectory followed by UAV when using estimated target information.

Noise was added to the target position in the form of measurement noise ($N(0,\;1.5^{2})$) and to target velocity in the form of process noise ($N(0,\;0.4^{2})$), both were considered to be non correlated and zero mean white Gaussian noise. Figure 9a,b show the estimated target trajectory and the simulated trajectory followed by the UAV to land on the target. UAV was able to land on the target in the presence of noise while satisfying the desired terminal conditions.

## 4. Experimental Results

We use an off-the-shelf quadcopter (3DR SOLO) for hardware validation of our landing algorithm. The landing algorithm runs on a Linux-based embedded computer–Odroid U3+. This is mounted at the bottom of the quadrotor and connected to a tilted Ptgrey chameleon USB camera. In addition to the camera, we also use a Sf02 Lidar connected to the Odroid via USB for better altitude estimates. These altitude estimates are used only by our controller running on odroid. Also, although a Lidar was mounted on the drone for better altitude estimates, this altitude estimate was not dependent on the presence of the target but rather the height of the drone above ground. Thus, though this altitude estimate helped in better estimate of target parameters this information by itself was not a result of active communication between the UAV and the target.

**Figure 10:**
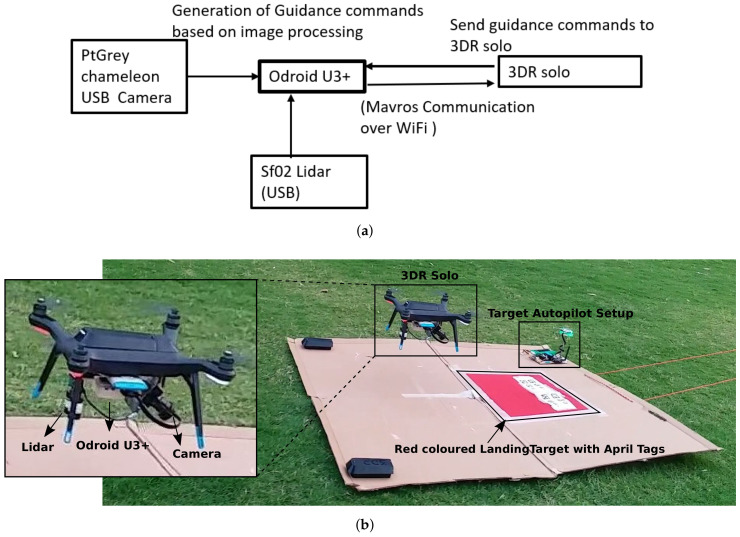
(**a**) Hardware setup and information flow diagram consisting of 3DR Solo (quadrotor), Odroid U3+, and PtGrey USB camera; (**b**) quadcopter with whole hardware interface about to land on target. Inset Figures show 3DR solo setup and detected target from onboard camera.

Output of our controller is in the form of velocity commands that are sent to the autopilot, where corresponding attitude commands are generated by the low level control loops in the autopilot. These attitude commands are finally converted to motor signals. Details of the hardware setup and the information flow are shown in Figure 10a. The proposed landing methodology is implemented using ROS [49], and OpenCV [50] is used to implement target detection. Figure 10b shows the quadrotor above the landing target. The inset Figures show 3DR solo with all the associated accessories and image obtained from the onboard camera, with the landing target detected by the vision algorithm. The communication between the onboard Linux computer (odroid) and the drone’s autopilot was established by connecting odroid to 3DR SOLO’s own WiFi network. Sensor data from the autopilot (drone’s compass heading etc.) is received by the onboard computer and velocity commands generated from the onboard computer are sent to the drone using this WiFi link. The onboard computer sends and receives information in the form of ROS messages, whereas the drone’s autopilot exchanges information in mavlink format. Thus, we use a standard ROS package *mavros* that acts as a bridge for message format conversion from ROS to mavlink and vice versa, thus establishing seamless exchange of information between the drone and the onboard computer. The videos of the landing experiments are available at Supplementary Materials.

### 4.1. Landing System Architecture

**Figure 11:**
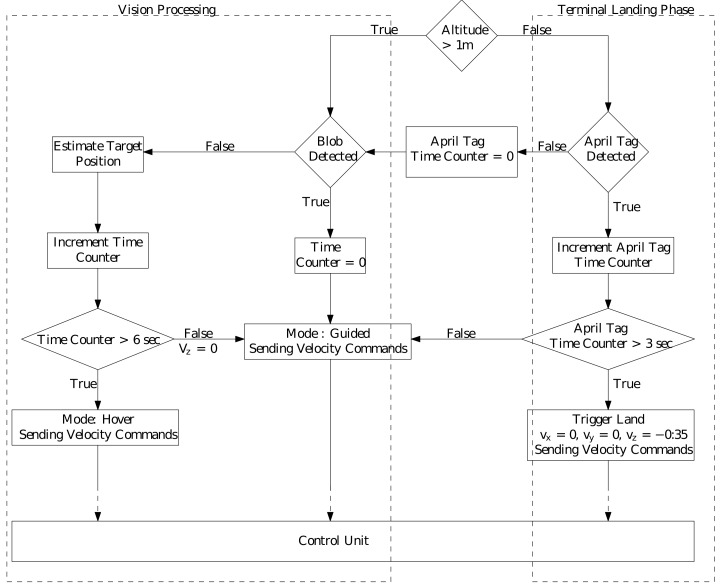
Vision and control pipeline for autonomous landing.

Landing system architecture consists of vision and control pipelines as shown in Figure 11. Vision processing pipeline is composed of detection and estimation submodules. Detection module consists of a combination of blob detection and AprilTag detection as explained in Section 2.1 and the estimation module consists of a Kalman Filter. Whenever the target is detected, appropriate velocity commands are published by the controller to the autopilot based on vision information. If no measurement is received by the estimator (target is not detected), guided velocity commands are published based on the target position predicted by the estimator, and a no-measurement time counter is started. If the target is not detected continuously for 6 s, $V_{x}=0,V_{y}=0,\;\mathrm{and}\;V_{z}=0$ velocity commands are published for x,y,z resulting in a state of hover until the target is detected again. The value 6 s was selected after several experiments. This transition of mode prevents any crashes or undesired maneuvers in cases of target occlusion and/or target going out of the field of view.

Next, we discuss the *Trigger Landing* logic during the terminal landing phase. As mentioned in Section 2.1, the camera footprint decreases with altitude and becomes very small during the final landing stage. Therefore, it is challenging to keep the entire blob in the camera frame, and thus only a part of the landing target might be visible. In such a scenario, only blob detection may not be sufficient to accurately detect and land on the target center. To this end, we place a set of 16 AprilTags of varying size inside the red colored landing target and the vision logic switches from blob detection to AprilTag detection below 1 m altitude. Multiple AprilTags with varying sizes make it easier to detect at least one AprilTag to aid the *Trigger Landing* process, during the final stage. Additionally, AprilTags are translation, rotation, scale, shear, and lighting invariant, resulting in robust detection. Below 1 m altitude, if at least one AprilTag is detected, it is given preference over the detected blob and AprilTag center is output by the vision pipeline as the landing target center. In the case of multiple AprilTag detection, estimator tracks the AprilTag selected in the previous frame (using the tagID information), for a smooth motion. If none of the detected AprilTags IDs match, the ID of tag detected in the previous frame, the vision algorithm does a random selection of one of the tags as the target center. During the trigger landing process, if at least one AprilTag is detected in every frame continuously for 3 s, then the controller triggers the landing action giving zero velocities in lateral and longitudinal direction and high value of downward *z* velocity (to compensate for ground effect). After touchdown, the vehicle is taken into manual control and disarmed. This approach of allowing the vehicle to touchdown followed by disarming and motor shutdown was an experimental design decision, as shutting off the motors directly below a threshold altitude can sometimes result in toppling of the vehicle and/or harm to onboard equipment.

### 4.2. Landing on Moving Target

Next, we evaluate the log polynomial controller for a moving target scenario. We conducted a total of 27 experiment runs to test the efficacy of the proposed strategy. The considered experiment cases included: landing on a target moving in a straight line, random trajectory and landing on a moving target with temporary midflight full target occlusion. We conducted 9 trials each for first two cases, and for the last case of mid flight full target occlusion, 5 trials with straight-line moving target and 4 trials with target moving in random trajectory were conducted. Details of these trials are presented in Section 5.

#### 4.2.1. Straight Line Target Motion

For this case, the quadcopter was commanded to takeoff to an altitude of 10 m and the target was moved in an approximate straight line motion with an approximate velocity of 0.3 m/s (as given by the autopilot mounted on the target) in the longitudinal direction and negligible movement in the lateral direction. In case of a moving target tracking and landing, the velocity of the UAV must be controlled according to the velocity of the target. Thus, it is important to compute the target velocity correctly. We use a KF for a robust estimate of the landing pad parameters $\left(x_{img},y_{img}\right)$, to deal with sudden changes in detection output, resulting from wind disturbances and target occlusion. The relative velocity on the image frame is calculated by the slope of the image positions. We can express the target velocity as given below:

(12) $$\left[\begin{array}{c} v_{\left(tx\right)}\\ v_{\left(ty\right)} \end{array}\right]=\left[\begin{array}{c} v_{qx_{t-\delta (t)}}\\ v_{qy_{t-\delta (t)}} \end{array}\right]+\left[\begin{array}{cc} K_{x} & 0\\ 0 & K_{y} \end{array}\right]\left[\begin{array}{cc} \cos(\psi ) & -\sin(\psi )\\ \sin(\psi ) & \cos(\psi ) \end{array}\right]\left[\begin{array}{c} \dot{x_{img}}\\ \dot{y_{img}} \end{array}\right].$$

where $\left(K_{x},K_{y}\right)$, represent the constants for transformation between the local NED coordinate and the image plane. The computed target velocity components are used to design the lateral and longitudinal velocity control subsystems independently using the log polynomial velocity profile. Altitude control was achieved using the velocity profile given in Equation (11), since the requirement in *z* direction during the terminal landing phase is $V_{z}\to 0,\mathrm{as}\;z\to 0$. For xy plane velocity control, since the aim is to drive the UAV target’s relative velocity to zero velocity, the profile remains the same as mentioned in Equations (5)–(7). Further, a flag was set for the quadrotor to descend such that quadrotor was commanded a *z* velocity only if this flag was equal to 1 as given in Equations (13)–(15),

(13) $$\begin{array}{ccc} flag_{descend} & = & 1,\mathrm{if}{\mathrm{flag}}_{\mathrm{detected}}=1, \end{array}$$

(14) $$\begin{array}{ccc} V_{z} & = & V_{z0}\log_{n}\left(1+ax+bx^{2}\right),\mathrm{if}{\mathrm{flag}}_{\mathrm{descend}}=1, \end{array}$$

(15) $$\begin{array}{ccc} V_{z} & = & 0,\mathrm{if}{\mathrm{flag}}_{\mathrm{descend}}=0. \end{array}$$

**Figure 12:**
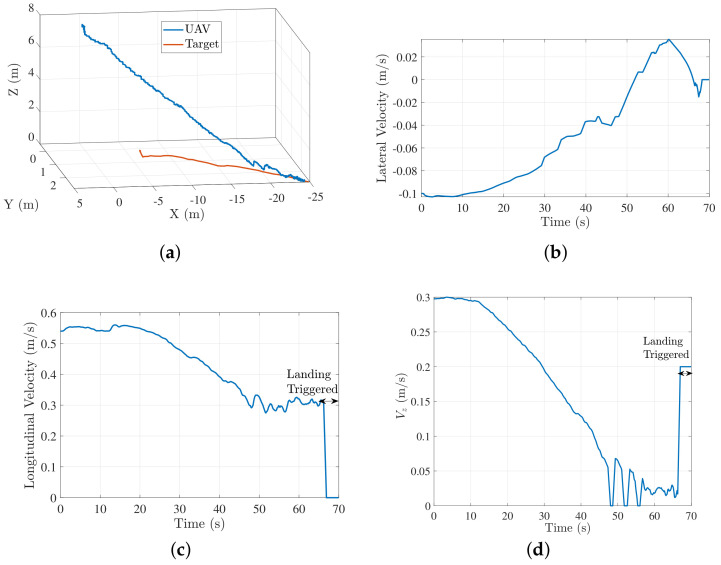
Trial 1: trajectory and velocity profile characteristics for moving target landing. (**a**) Trajectory followed by the quadcopter to land on a moving target; (**b**) lateral velocity profile (a,b,c)=(10,130,−131); (**c**) longitudinal velocity profile (a,b,c)=(40,130,−161) (**d**) $V_{z}$ profile (a,b)=(6,3).

The flag was set to 1, if the target was detected by the vision system. In case of no target detection, commands for tracking the target based on estimates were published. We conducted several trials to evaluate the efficacy of our controller. Details of these experiments are presented in Section 5. Next, we discuss the results from 2 trials (trial 1 and 2) for landing on a target moving in a straight line. Figure 12a shows the trajectory followed by the quadrotor to land on the target during trial 1. Figure 12b–d show the longitudinal, lateral and descend velocity profiles of the quadrotor respectively for this trial. The longitudinal velocity profile approaches the target speed toward the end and the lateral velocity profile approaches 0, thus resulting in zero closing velocity for a successful landing.

**Figure 13:**
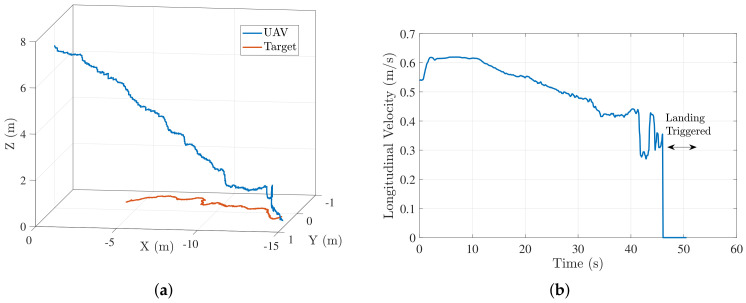
Straight line moving target trial 2: (**a**) vehicle’s trajectory; (**b**) longitudinal velocity profile for landing on a straight-line moving target for a different set of log polynomial parameters (a,b,c)=(55,115,−161).

**Figure 14:**
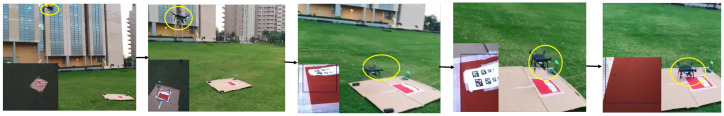
Landing sequence on a ground moving target.

Figure 13 shows results of trial 2 straight-line moving target . For this trial, a different set of log polynomial parameters were generated randomly ((a,b,c)=(55,115,−161)). Initial conditions for this experiment (UAV-target distance, initial UAV velocity, target velocity) were same as that of trial 1. Figure 13b shows that a faster landing is achieved in trial 2. This is because of a higher value of log polynomial parameter *a* in trial 2 (a=55) resulted in a higher initial acceleration compared to that of trial 1 (a=40). We show only the longitudinal velocity profile for trial 2 as significant motion of target is in longitudinal direction only. Figure 14 shows the external camera view of a typical landing sequence. Inset figure in every frame shows the onboard camera view.

#### 4.2.2. Target Moving in a Random Trajectory

**Figure 15:**
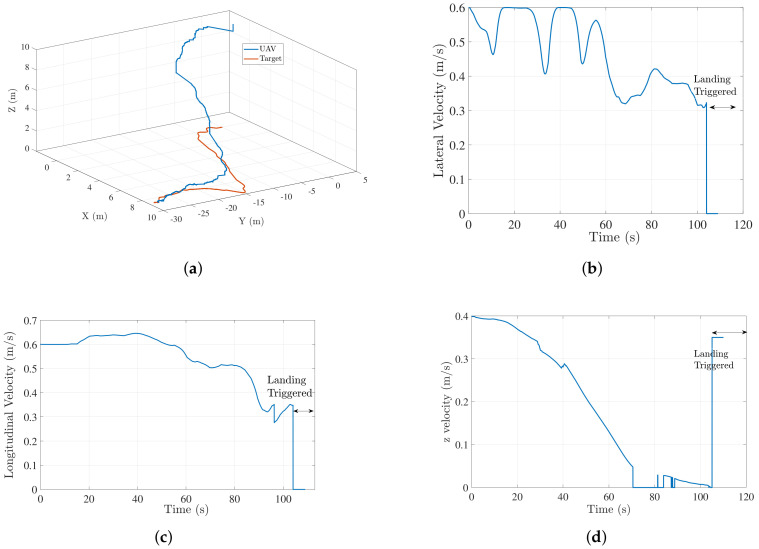
Case1: (**a**) UAV-target trajectory for a nonstraight-line moving target landing; (**b**) lateral velocity profile of UAV (a,b,c)=(40,130,−161); (**c**) longitudinal velocity profile of UAV (a,b,c)=(40,130,−161) (**d**) z velocity profile of the UAV (a,b)=(6,3).

**Figure 16:**
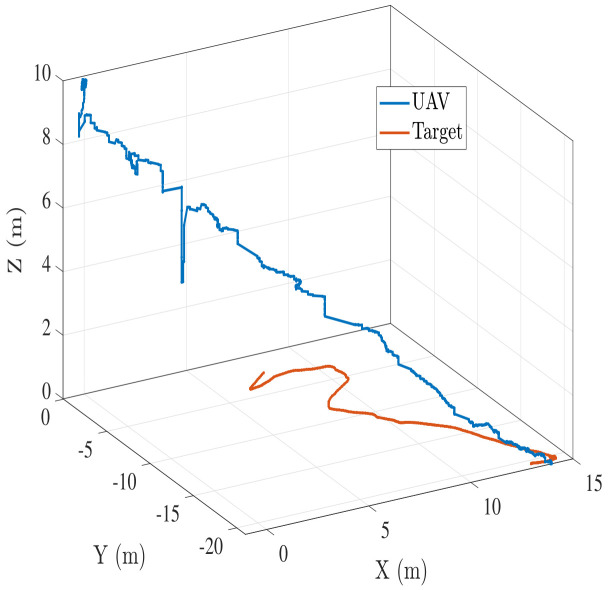
Case2: UAV-target trajectory for a nonstraight-line moving target landing.

In addition to a straight-line moving target, we also performed hardware experiments for target moving in a random trajectory with an approximate speed of 0.4 m/s. Figure 15a shows the UAV-target trajectory for such a scenario. Figure 15b–d show the lateral, longitudinal, and z velocity profiles respectively for the trajectory in Figure 15a. In both cases, UAV is able to land successfully on the target with UAV velocity eventually converging to target velocity before the landing is triggered. Figure 16 shows another scenario for a nonstraight-line moving target landing. The altitude jump in Figure 16 is attributed to the spike in LIDAR data due to a slight change in ground slope.

#### 4.2.3. Landing on a Moving Target with Midflight Occlusion

One of the major challenges of the landing process is losing the sight of the target. This can happen if the target gets partially or fully occluded due to obstacles and/or goes out of field of view. It results in a deviation from the desired trajectory. To overcome this situation, we use the KF to achieve a robust estimate of the landing pad parameters. The output of detection algorithm ($x_{t}$ and $y_{t}$ centroid pixel coordinates of the target) are given as input to the estimator. The extracted target parameters using vision are used to generate appropriate guidance commands for the quadrotor.

**Figure 17:**
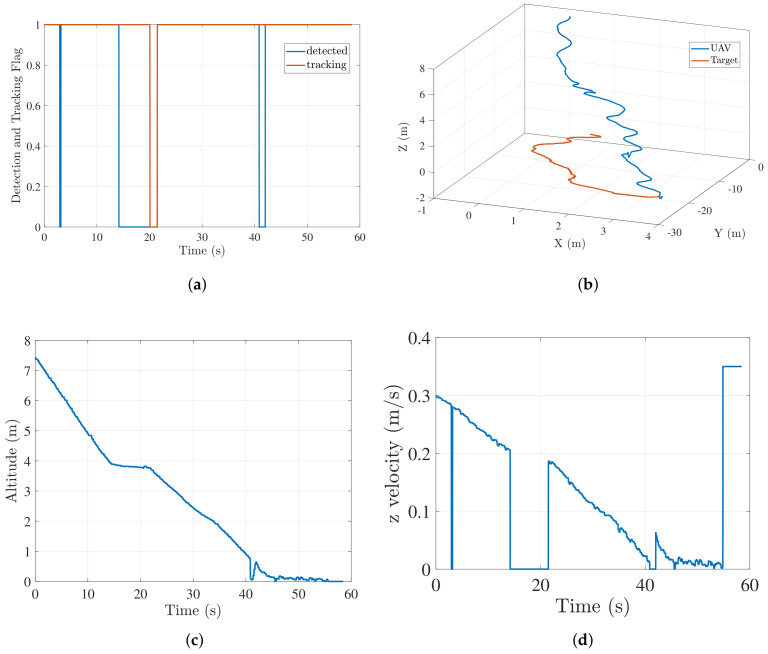
Moving target occlusion results. (**a**) Target detected and tracking flag; (**b**) trajectory followed by UAV to land on target; (**c**) altitude profile of UAV; (**d**) $V_{z}$ profile of UAV.

For this experiment case, we fully occluded the target during certain phases of the flight. Figure 17 shows the results for one of the instances where the target was manually occluded. In Figure 17a, a zero value of detected flag indicates that the target is occluded and/or out of field of view. When the target is occluded, no measurements are received, and the predicted estimate by KF is used for 6 s (empirically chosen) to generate the guidance commands. This is represented by the value of 1 for tracking flag even when the detection flag is 0. After 6 s, zero velocity commands are published by the controller and tracking flag is set to 0, until the landing pad is detected again. Figure 17b shows the trajectory followed by the UAV to land on the target in midocclusion flight. Since the target was recovered after the occlusion, the vehicle is able to land on the target successfully.

Figure 17c,d show the altitude and z velocity profile, respectively. Whenever the detection flag is zero, $V_{z}$ is also zero, thus maintaining a nearly constant altitude that results in a nondecreasing camera footprint, making the target reacquisition easier. The second instance of target leaving UAV camera view (just after the 40 s time mark) shows a sudden jump in altitude in Figure 17c. Although the corresponding commanded *z* velocity in Figure 17d is zero. The sudden spike in altitude was the result of an atypical behavior of the autopilot due to commanded *z* velocity being zero momentarily at very low altitude. For this specific case, we were able to reacquire the target because of the altitude spike. In the future, we plan to incorporate a hybrid approach for similar scenarios. As a part of the hybrid approach, we plan to include a search strategy where, if the target is lost for more than a prescribed threshold period, the controller will command an increase in UAV altitude to aid search, and hence, the target reacquisition.

## 5. Discussion

As discussed in Section 1, significant work was performed in literature for autonomous landing of a quadrotor on moving targets. In [27] authors proposed a guidance-based approach for autonomous landing. The approach consisted of PN (proportional-navigation) guidance during the approach phase augmented with a closing velocity controller and proportional-derivative (PD) controller for the final landing phase. However, the closing velocity controller was not tested with vision in the loop and was only limited to approach phase with UAV flying at a constant altitude. Such a closing velocity controller may not work for landing when there is a simultaneous decrease in altitude. Although experiments showed landings at higher target speeds, their approach relied on target information from inertial/global positioning system(GPS) sensors placed on the ground vehicle, in addition to vision. In [51], the tracking guidance for the vision-based landing system was based on relative distance between the UAV and the target. However, the decrease in altitude during the landing process was a *step-wise* decrease, i.e., a downward velocity was commanded intermittently only when the UAV was directly above the target and UAV was in target tracking mode (at constant altitude) at other times. Such a noncontinuous decrease contributed to an increase in total landing time. This is one of the examples that highlights the advantage of our approach, where a common log polynomial speed controller function (albeit with different parameter values) for horizontal and vertical speed control provides explicit control over the speed profile characteristics resulting in a time-efficient landing. From an evaluation perspective, most approaches in literature reported the results in the form of aggregate landing accuracy from a limited number of trial runs [7,27,52]. We improve on the existing work through a more extensive evaluation. We conduct a total of 27 outdoor field experiments consisting of straight line as well as random target trajectory cases (with and without intermittent target occlusion). Further, we report the average landing time of each trial category in addition to landing accuracy. Next, we present the details of the experiment runs along with the observations and lessons learned from failure landing cases.

**Figure 18:**
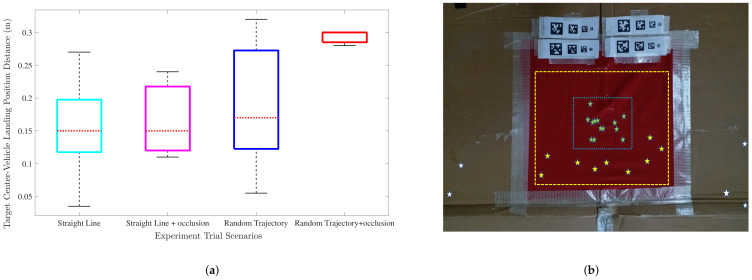
(**a**) Box plot of successful experiment trials for different target trajectory cases. (**b**) Depiction of vehicle landing position in different experiment runs.

The criteria to evaluate whether a trial was successful was the distance of the vehicle from the target center. A trial with less than |40| cm distance between the vehicle’s final landing position and target center was categorized as successful; otherwise, unsuccessful. Table 1 shows the categorization of experiment trials along with the details of landing accuracy and the number of unsuccessful trials. As per the criteria mentioned above, we were able to achieve a successful landing in 22 runs. In 13 of these runs, we were able to achieve a landing within ±20 cm from the landing target center. For the rest of the 9 experiments, the landing was achieved within ±35 cm of the target center. The table also shows the average landing time and best landing accuracy (least error between vehicle landing position and target center) achieved in each category. During the successful trials, the fastest landing was achieved in 41 s in the straight-line moving target scenario with the log polynomial parameter values of (a,b,c) = (61,109,−161) with a landing accuracy of |0.12| m. Maximum and average UAV speed for this case in the direction of target motion was 0.67 m/s and 0.5 m/s, respectively. Log polynomial parameter values of a>61, although provided a higher initial acceleration of UAV velocity resulting in a faster landing, the landing accuracy suffered. This was in all likelihood due to a rapid deceleration and ungraceful decay of closing velocity during the final stages of the landing process. Figure 18a shows a boxplot for successful experiment trials. Y-axis represents the observed distance between target center and vehicle landing position during these trials and the 4 boxes correspond to the four experiment scenarios discussed in Table 1. The boxplot shows that the median of error for successful landing trials is well below 0.2 m for all the cases, except random trajectory with target occlusion where it is 0.30 m. Figure 18b depicts the vehicle landing position for different experiment runs. Green markers and yellow markers show the cases for vehicle landing within ±20 and ±35 cm from the landing target, respectively. White markers represent the failure cases where the vehicle failed to land on the target.

We had 5 unsuccessful runs where the vehicle landed away from the target. Among the unsuccessful trials, the maximum observed vehicle distance from the target was 0.6 m, which occurred in the “random trajectory moving target with mid-flight occlusion” case. During this trial, occlusion was introduced at approximately 4 m of altitude. Additionally, the trajectory of the moving target was perturbed, while the target was occluded. Thus estimator (in the absence of measurements) was unable to predict the target motion correctly during the occlusion phase.

It was observed during the trials that all the failures occurred due to target going out of field of view, because of (i) erroneous estimation of target motion at low altitudes during occlusion and (ii) perturbations in target trajectory, especially at the time when quadcopter was slightly lagging behind the target. These failures can be attributed to an increase in the relative estimation error of the KF at lower altitudes (because of a decrease in camera footprint). Instances of the vehicle lagging behind the target due to estimation errors were also observed during successful trials. However, the controller was robust and quick enough to catch up as soon as the target was detected, and the state was updated correctly.

For all the experiments, a strictly decelerating profile was chosen for *z*-direction since a smooth decrease in camera footprint is desirable for maintaining target visibility. Log polynomial parameters for lateral and longitudinal velocity profiles were initialized based on the estimated target motion. For example, for the straight-line moving target case, an initially accelerating profile followed by deceleration was chosen for longitudinal direction, since the significant target motion was limited only to this direction only. In the case of random target trajectory landing, an initially accelerating profile was chosen for lateral as well as longitudinal direction since the target had significant motion in both the directions.

From our experiment trials, we inferred that any environment disturbances such as wind, or any abrupt changes in target trajectory results in target going out of the camera view. To this end, we evaluated conditions for descending velocity to maintain target visibility more consistently.

**Figure 19:**
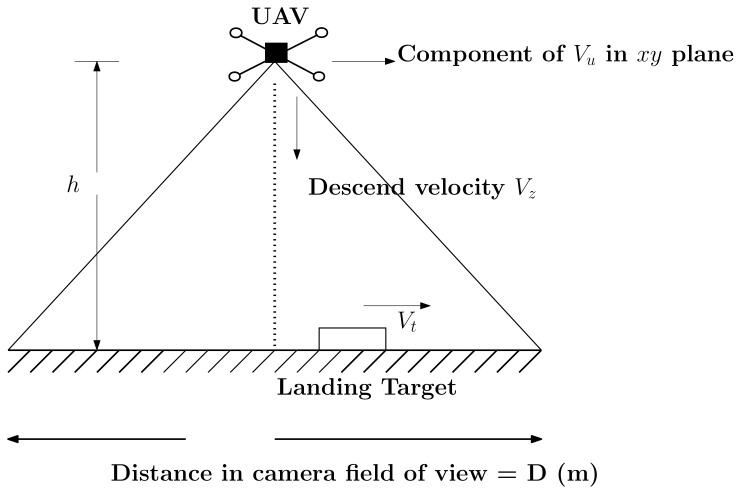
A depiction of a quadrotor trying to land on a moving target with velocity $V_{t}$.

Consider an aerial scenario as shown in Figure 19, where a quadrotor (with a velocity $V_{u}$), and with a downward facing camera, is attempting to land on a moving target with velocity $V_{t}$. At time *t*, quadrotor is at a distance *R* from the landing target and an altitude of *h* meters above the ground.

Let *D* meters be the distance (in meters) present in the camera field of view at a height *h* above the ground at time *t*. The condition of keeping the target in field of view can be expressed as, $R\leq D,\dot{R}<0$. Given such a scenario, the target remains within the field of view of the UAV, as long as the velocity of UAV is higher and/or consistent with the target velocity and a near zero relative velocity is maintained in the final stages of landing. With known intrinsic camera parameters and quadrotor velocity profile, a bound in descend velocity can be calculated so as to maintain target visibility.

Given camera parameters (field of view in degrees), and log polynomial controller given by Equation (5), the condition for maintaining target visibility can be expressed as:

(16) $$\begin{array}{c} V_{z}<\frac{2*V_{t}*\left(N-1\right)}{\tan(\frac{fov}{2})}, \end{array}$$

where fov is field of view of the camera in degrees, *N* is the ratio of initial UAV to target speed, $V_{z}$ and $V_{t}$ is descend velocity and target speed, respectively. The current experiment trials were performed at low target speeds. While we would have liked to test our controller at higher speeds, a combination of factors like our system configuration, along with the added payload and safety restrictions of the available outdoor experiment space, constrained us to limit our experiments to lower speed moving targets.

In addition to driving the closing velocity to zero and achieving a good landing accuracy, one of the significant challenges for landing on a fast-moving target is to maintain its visibility consistently. Based on the lessons learned from our experimental trials, for landing on a higher speed moving target using our proposed framework, we anticipate more challenges for circular and random target trajectories (with and without occlusion) than a straight-line target trajectory.

For the scenarios mentioned above, a robust estimation of target motion is generally one of the major concerns. For example, in our case, the camera frame rate of 15 Hz is most likely too less for a target moving in a random trajectory with a speed of 10 m/s. Since our estimate relies on continuous target measurements to correct its estimate, prediction uncertainties would be higher with a low update rate of target measurements and much higher during target occlusion. Although in our previous work we performed an extensive analysis of different velocity profiles with variation in log polynomial parameters, a choice of these parameters for landing on a high-speed moving target in the presence of estimation errors would be another challenge, as any sudden change at high aerial vehicle speed is likely to cause aggressive motion of the drone. Thus, we intend to develop an automated method for log polynomial parameter selection during the landing process and use the result presented in Equation (16) to further improve the robustness of our approach for landing on higher speed moving targets.

## 6. Conclusions

In this paper, a guidance-based landing approach is presented for autonomous quadrotor landing with vision in the loop. A log-polynomial closing velocity controller is developed and integrated with pure pursuit (PP) guidance for a time-efficient landing with minimum closing velocity. The integrated approach enables the vehicle to land faster compared to that of the traditional track and vertical landing. The efficacy of the proposed approach is validated exhaustively through simulations. We also perform extensive outdoor field experiments to validate the proposed approach for a stationary and moving landing pad using a quadrotor with onboard vision. The results show that the quadrotor is able to land on the target under different environmental conditions. The landing experiments were carried in mild wind conditions. To explore the ability of the quadrotor to land under high wind conditions, one may have to design observers and nonlinear controller that are robust to these disturbances. A potential future work involves a more sophisticated mechanism of choice of controller parameters at the beginning and midflight to deal with high speed, as well sudden accelerations/change in target motion. Another direction of work could be to develop novel landing controllers for ship-based landing where the target has a 3D motion and the environment has wind disturbances, which are a function of the sea state. One could also explore different sensor fusion approaches for landing and show a comparison of different approaches for landing.

## Figures and Tables

**Table 1:** Categorization of experimental trials according to target trajectory and target occlusion.

Target Trajectory	No. of Trials	Final Vehicle Distance from Target Center for Successful Trials (in M)	Unsuccessful Landings (Failure Cases)	Best Landing Accuracy	Average Landing Time
Straight line trajectory, no occlusion	9	7 trials ≤0.20, 2 trials ≤0.35	None	0.035 m	56.5 s
Straight line trajectory, with occlusion	4	2 trials ≤0.20, 1 trial ≤0.35	1 with an absolute error of 0.47 m	0.11 m	1 min 2 s
Random trajectory, no occlusion	9	4 trials ≤0.20, 3 trials ≤0.35	2 with absolute errors 0.45 m and 0.54 m respectively	0.055 m	1 min 38 s
Random trajectory, with occlusion	5	3 trials ≤0.35	2 with absolute errors 0.60 m 0.55 m respectively	0.25 m	1min 37 s

**Figure A1:**
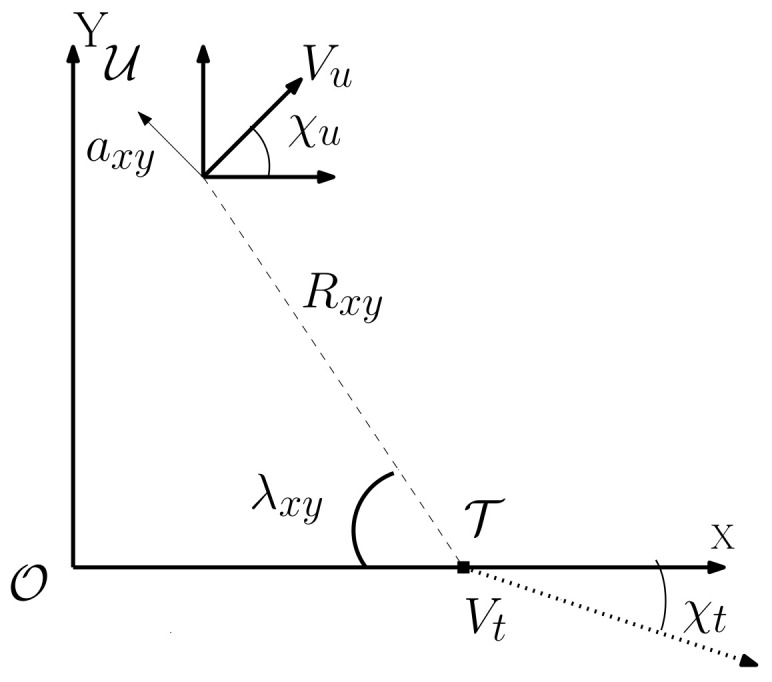
Planar engagement geometry of UAV target.

**Figure A2:**
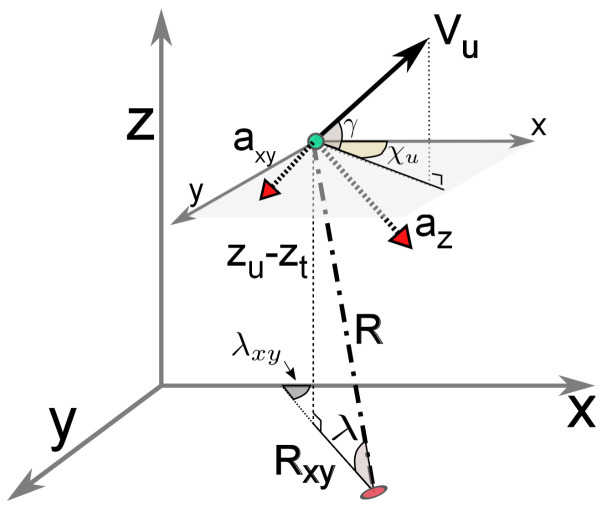
UAV target engagement geometry for 3D landing process.

## Data Availability

Not applicable.

## Supplementary Materials

The following supporting information can be downloaded at: https://www.mdpi.com/article/10.3390/s22031116/s1.

## Appendix A. Basics of Pure Pursuit (PP) Guidance

In this section, we provide an overview of 2D and 3D pure pursuit guidance laws for completeness, and then use these guidance laws for landing in the subsequent sections.

### Appendix A.1. 2D Pure Pursuit (PP) Guidance

Consider a planar UAV-target engagement geometry as shown in Figure A1. The speed of the UAV and the target is $V_{u}$ and $V_{t}$, respectively. The notation $\chi_{u}$, $\chi_{t}$, $\lambda_{xy}$, and $R_{xy}$ represent the UAV course angle, target course angle, line-of-sight (LOS) angle, and the distance between target and the UAV (range), respectively. The target is assumed to be nonmaneuvering; that is, $\chi_{t}$ is constant. Using the engagement geometry in Figure A1, ${\dot{R}}_{xy}$ and ${\dot{\lambda }}_{xy}$ are calculated as,

(A1) $$\begin{array}{ccc} {\dot{R}}_{xy} & = & V_{t}\cos\left(\chi_{t}-\lambda_{xy}\right)-V_{u}\cos\left(\chi_{u}-\lambda_{xy}\right), \end{array}$$

(A2) $$\begin{array}{ccc} R_{xy}{\dot{\lambda }}_{xy} & = & V_{t}\sin\left(\chi_{t}-\lambda_{xy}\right)-V_{u}\sin\left(\chi_{u}-\lambda_{xy}\right). \end{array}$$

The guidance command, denoted as $a_{xy}$ is the lateral acceleration applied normal to the velocity vector. The guidance command is expressed as,

(A3) $$\begin{array}{cc} {\dot{\chi }}_{u} & =a_{xy}/V_{u}. \end{array}$$

The principle of PP is to align $\chi_{u}$ towards $\lambda_{xy}$ [53], so that the UAV velocity vector is directed towards the target. A practical implementation of the pure pursuit uses a feedback law as,

(A4) $$\begin{array}{cc} a_{xy} & =-K_{a}\left(\chi_{u}-\lambda_{xy}\right),K_{a}>0, \end{array}$$

where $K_{a}$ is the gain. However, for PP to be effective, it is important to maintain ${\dot{\chi }}_{u}$=${\dot{\lambda }}_{xy}$, so that the vehicle can point towards the target at all times. Combining Equations (A3) and (A4), the guidance command for simple pure pursuit is given as,

(A5) $$\begin{array}{cc} a_{xy} & =V_{u}{\dot{\lambda }}_{xy}-K_{a}\left(\chi_{u}-\lambda_{xy}\right). \end{array}$$

The simplicity of implementation and this inherent property of PP guidance to point at the target at all times makes it an ideal choice for vision-based landing applications, as it ensures persistent target detection and tracking as the vehicle closes in for landing.

### Appendix A.2. 3D Pure Pursuit Guidance

We extend the 2D pursuit guidance principle to 3D for landing. We consider the target moving on XY horizontal plane. Assume that the initial location of the UAV is ($x_{0}$, $y_{0}$, $z_{0}$) and the target is visible from this initial location. Figure A2 shows the engagement geometry for a UAV landing on the target in a 3D scenario. As the landing is a 3D process, we need the UAV course angle $\chi_{u}$ and flight path angle γ information. Expressions for UAV target distance, LOS angle λ, and projection of LOS angle in XY plane $\lambda_{xy}$ are given as,

(A6) $$\begin{array}{ccc} R & = & \sqrt{{\left(x_{t}-x_{u}\right)}^{2}+{\left(y_{t}-y_{u}\right)}^{2}+{\left(z_{t}-z_{u}\right)}^{2}}, \end{array}$$

(A7) $$\begin{array}{ccc} R_{xy} & = & \sqrt{{\left(x_{t}-x_{u}\right)}^{2}+{\left(y_{t}-y_{u}\right)}^{2}}, \end{array}$$

(A8) $$\begin{array}{ccc} R_{z} & = & z_{t}-z_{u}, \end{array}$$

(A9) $$\begin{array}{ccc} \lambda_{xy} & = & \tan^{-1}\left(y_{t}-y_{u}/x_{t}-x_{u}\right), \end{array}$$

(A10) $$\begin{array}{ccc} \lambda & = & \tan^{-1}\left(z_{t}-z_{u}/R_{xy}\right). \end{array}$$

Expressions of ${\dot{R}}_{xy}$, $\dot{R_{z}}$, ${\dot{\lambda }}_{xy}$ are derived similar to the 2D case.

(A11) $$\begin{array}{ccc} {\dot{R}}_{xy} & = & V_{t}\cos\left(\chi_{t}-\lambda_{xy}\right)-V_{u}\cos\gamma \cos\left(\chi_{u}-\lambda_{xy}\right), \end{array}$$

(A12) $$\begin{array}{ccc} {\dot{R}}_{z} & = & V_{u}\sin\gamma , \end{array}$$

(A13) $$\begin{array}{ccc} R_{xy}{\dot{\lambda }}_{xy} & = & V_{t}\sin\left(\chi_{t}-\lambda_{xy}\right)-V_{u}\cos\gamma \sin\left(\chi_{u}-\lambda_{xy}\right). \end{array}$$

Guidance command in 3D is given by,

(A14) $$\begin{array}{ccc} a_{xy} & = & V_{u}\cos\gamma {\dot{\lambda }}_{xy}-K_{a}\left(\chi_{u}-\lambda_{xy}\right), \end{array}$$

(A15) $$\begin{array}{ccc} a_{z} & = & V_{u}\dot{\lambda }-K_{a}\left(\gamma -\lambda \right), \end{array}$$

(A16) $$\begin{array}{ccc} {\dot{\chi }}_{u} & = & a_{xy}/\left(V_{u}\cos\gamma \right), \end{array}$$

(A17) $$\begin{array}{ccc} \dot{\gamma } & = & a_{z}/V_{u}. \end{array}$$

We assume the target is nonmaneuvering and traveling with a constant velocity and heading. The kinematic model for the target and UAV are given as

(A18) $$\begin{array}{ccc} x_{t_{t+\Delta t}} & = & x_{t_{t}}+V_{t}\cos\chi_{t}\Delta t, \end{array}$$

(A19) $$\begin{array}{ccc} y_{t_{t+\Delta t}} & = & y_{t_{t}}+V_{t}\sin\chi_{t}\Delta t, \end{array}$$

(A20) $$\begin{array}{ccc} x_{u_{t+\Delta t}} & = & x_{u_{t}}+V_{u}\cos\gamma \cos\chi_{u}\Delta t, \end{array}$$

(A21) $$\begin{array}{ccc} y_{u_{t+\Delta t}} & = & y_{u_{t}}+V_{u}\cos\gamma \sin\chi_{u}\Delta t, \end{array}$$

(A22) $$\begin{array}{ccc} z_{u_{t+\Delta t}} & = & z_{u_{t}}+V_{u}\sin\gamma \Delta t. \end{array}$$

## Appendix B. Proof: Convergence of Closing Velocity to Zero Using Log Polynomial Controller

**Theorem** **A1.**
*Let f(x) be a log of polynomial function of the ratio of current to initial line of sight distance ($R_{xy}/R_{xy}^{0}$). Then, the closing velocity ($-{\dot{R}}_{xy}$) asymptotically decreases to zero as the distance goes to zero if $V_{u}$=$f\left(x\right)V_{t}$.*

**Proof.** In PP, the aim is to continuously point towards the target and the guidance command is aimed at maintaining $\chi_{u}$=$\lambda_{xy}$. Therefore Equation (A1) reduces to

(A23) $$\begin{array}{ccc} {\dot{R}}_{xy} & = & V_{t}\cos\left(\chi_{t}-\lambda_{xy}\right)-V_{u}. \end{array}$$

As, the UAV approaches the target, $R_{xy}$→ 0. In PP as $R_{xy}\to 0$, it becomes a tail chase scenario where the UAV is just behind the target and $\chi_{u}\approx \chi_{t}$. But since PP inherently maintains $\chi_{u}\approx \lambda_{xy}$ it can be deduced that as $R_{xy}\to 0$, $\lambda_{xy}\to \chi_{t}$, reducing Equation (A23) to

(A24) $$\begin{array}{c} \lim_{R_{xy}\to 0}{\dot{R}}_{xy}=V_{t}-V_{u}. \end{array}$$

The variation in velocity is given as,

(A25) $$\begin{array}{ccc} V_{u} & = & V_{t}f\left(x\right), \end{array}$$

where,

(A26) $$\begin{array}{ccc} N & = & V_{u}^{0}/V_{t}^{0}, \end{array}$$

(A27) $$\begin{array}{ccc} x & = & R_{xy}/R_{xy}^{0}. \end{array}$$

Initially, $R_{xy}$ = $R_{xy}^{0}$, hence *x* = 1. Therefore, from Equations (5) and (A25)

(A28) $$\begin{array}{ccc} \log_{n}\left(1+ax+bx^{2}+cx^{3}\right) & = & 1, \end{array}$$

(A29) $$\begin{array}{ccc} V_{u} & = & NV_{t}\;\;if\;\;R_{xy}=R_{xy}^{0}. \end{array}$$

As UAV approaches the target and $R_{xy}$→ 0, *x*→ 0 and $log_{n}\left(1+ax+bx^{2}+cx^{3}\right)$→ 0. As a result,

(A30) $$\begin{array}{ccc} \lim_{R_{xy}\to 0}V_{u} & \to & V_{t},\mathrm{and} \end{array}$$

(A31) $$\begin{array}{ccc} \lim_{R_{xy}\to 0}{\dot{R}}_{xy} & \to & 0. \end{array}$$

□
